# Edge-dependent superconductivity in twisted bismuth bilayers

**DOI:** 10.1039/d6na00532b

**Published:** 2026-10-07

**Authors:** Isaías Rodríguez, Renela M. Valladares, Alexander Valladares, David Hinojosa-Romero, Flor B. Quiroga, Ariel A. Valladares

**Affiliations:** a Instituto de Investigaciones en Materiales, Universidad Nacional Autónoma de México, Ciudad Universitaria Apartado Postal 70-360 CDMX 04510 Mexico; b Facultad de Ciencias, Universidad Nacional Autónoma de México, Ciudad Universitaria Apartado Postal 70-542 CDMX 04510 Mexico renelavalladares@ciencias.unam.mx

## Abstract

Twisted bilayers offer a platform for engineering new twistronic materials. Whereas standard studies focus on the enigma of twist-angles, epitomized by twisted graphene bilayers, functional devices face a fundamental concern: boundary heterogeneity. In this study, we address this by investigating the electronic and vibrational properties of twisted bismuth bilayer flakes. Twisted flakes exhibit interesting variations of these properties, away from the bulk, as we report herein using *ab initio* density functional theory, systematically mapping the evolution of the electronic density of states, the vibrational densities and possible superconductivity. Our work reveals a non-fortuitous consequence of structural disorder at the edges: an enhanced electronic density of states at the Fermi level. This enhancement reaches a maximum of 10 times that of crystalline bismuth. Given that the superconducting critical temperature is exponentially dependent on the electronic density of states, this structural variation suggests a mechanism for increasing *T*_c_. We identify the edge disorder as a parameter in designing devices with enhanced properties. Our results provide a theoretical framework for interpreting new twistronic data, paving the way to search for disordered metastable structures that could lead to enhanced superconducting transition temperatures.

## Abstract 2

Twisted bilayer bismuth flakes feature crystalline cores and disordered edges. These amorphous-like edges exhibit highly oscillatory electronic states, unlocking remarkable superconductivity with a peak *T*_c_ of 37.53 K at a 30° twist-angle.

## Graphical abstract

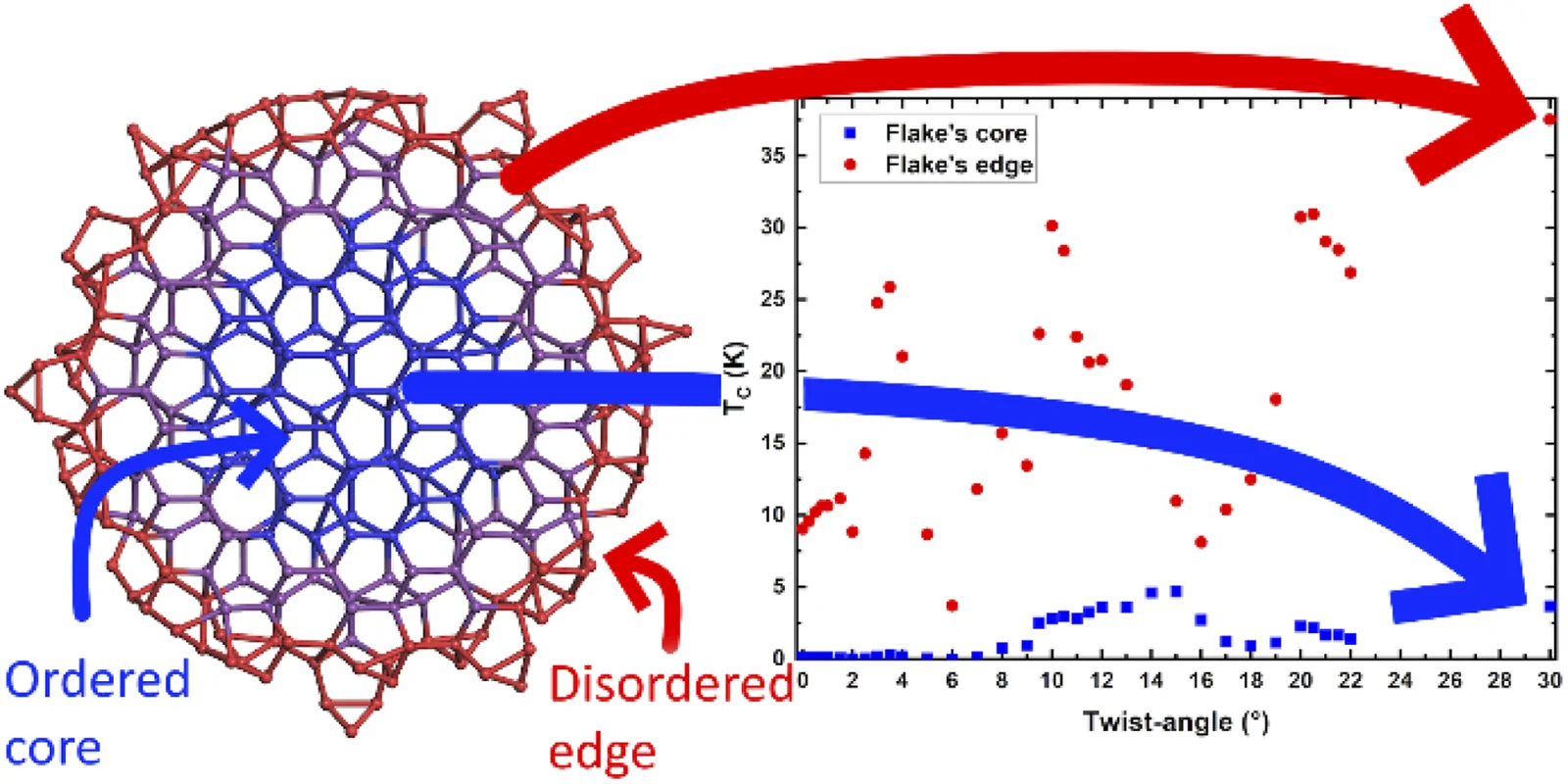

## 1 Introduction

Monolayer bismuthene, defined as a jagged single-atom allotrope of bismuth, already stands as a remarkable quantum material because of its bandgap (0.5 eV).^1^ Beyond theoretical fascination, these monolayers foster a potentially room-temperature quantum spin Hall effect^2^ and the existence of stable disordered edge states that have been experimentally reported.^3^ Nevertheless, the ultimate challenge in modern material science is not a single layer's capabilities, but how we can possibly design a structure by stacking these monolayers.

The groundbreaking discovery of twisted graphene bilayer (TGB) unveiled the fact that double-layer stacking introduces an extra degree of freedom, the twist-angle, that radically changes the electronic behavior of the material. Unexpected results, from superconductors to related insulators, are obtained by this interlayer coupling that gives rise to flat electronic bands at specific “magic” twist-angles (∼1.1°),^3–6^ due to the moiré patterns.^4^ However, the discipline faces a major obstacle: whereas early theoretical models employed ideal, unflawed periodicity,^7–11^ experimental results reveal a natural spatial heterogeneity in the flake's edges.^3,12^

In our recent work, for flawless flakes,^13^ we depicted the deconstruction of the perfect image of twisted bismuth bilayer (TBB). Mere achievement of global twist-angle homogeneity in a finite flake is insufficient to guarantee electronic homogeneity on the whole flake, due to the presence of border effects.

Atomistic simulations unequivocally demonstrate that local lattice relaxation and unavoidable strain gradients cause moiré period distortions, particularly near the flake boundaries. These topological effects are not artifacts; they are stable within created structures, forcing the inevitable conclusion that topological edge reconstruction plays a crucial role in device performance. We have to address this real-world structural disorder so we can actually engineer the electronic usefulness of these systems.

Despite previous results, still lurking at its heart is a persistent inhomogeneity: how accurately do the electronic states evolve at the TBB flake edges from its homogeneous bulk-like configuration as the flake relaxes and the twist-angle gradually increases? This local inhomogeneity, due to the generated topological disorder, is no mere casual defect; it is the generic structural issue of real devices.^14–17^ Experiments have already detected anomalous electronic signatures, including pseudogap suppression and the appearance of 1D channels,^18^ but there is still a missing link: a theory that connects these electronic phenomena directly with disorder and with the twist-angle.

Here, we bridge this critical gap by systematically investigating the electronic properties of the TBB core and edges under selected twist-angle variations. We use our well-established *ab initio* methodology, based on the density functional theory,^19^ to map the evolution of the electronic structure, the electronic density of states (eDoS, *N*(*E*)), and related properties across these distinctive arrangements. Our results deliver the key finding: a significant enhancement of the eDoS at the Fermi level (*N*(*E*_F_)) due to the flake borders, showing an increase of 10 times that of perfect crystalline bismuth. This large increment in the *N*(*E*_F_) dictates a mechanism for achieving an increase in the superconducting critical temperature (*T*_c_) by creating disordered metastable atomic structures.

## 2 Methods

We began with the crystalline Bi I Wyckoff structure,^20^ subjecting it to a substantial 20 × 16 × 2 multiplication to yield a large, yet necessary, 1160-atom supercell (Fig. 1a). This large scale was crucial to accurately capture the physics of the long-range moiré pattern and local twist-angle variations. From this initial supercell, we removed atoms to isolate two freestanding flakes. Each of these flakes contains 145 atoms, resulting in a non-optimized hexagonal structure containing 290 atoms (Fig. 1b). Then the flake was geometry-relaxed, and a total of thirty-three distinct moiré configurations were prepared. Each one was generated by rotating one flake relative to the other around the normal axis. The specific twist angles selected were 0.0° (Fig. 1c, optimized and non-rotated), 0.25°, 0.5°, 0.75°, 1.0°, 1.5°, 2.0°, 2.5°, 3.0°, 3.5°, 4.0°, 5.0°, 6.0°, 7.0°, 8.0° (Fig. 1d, optimized), 9.0°, 9.5°, 10.0°, 10.5°, 11.0°, 12.0°, 13.0°, 14.0°, 15.0°, 16.0°, 17.0°, 18.0°, 19.0°, 19.5°, 20.0°, 20.5°, 21.5°, 22.0°, and 30.0°.

**Fig. 1:**
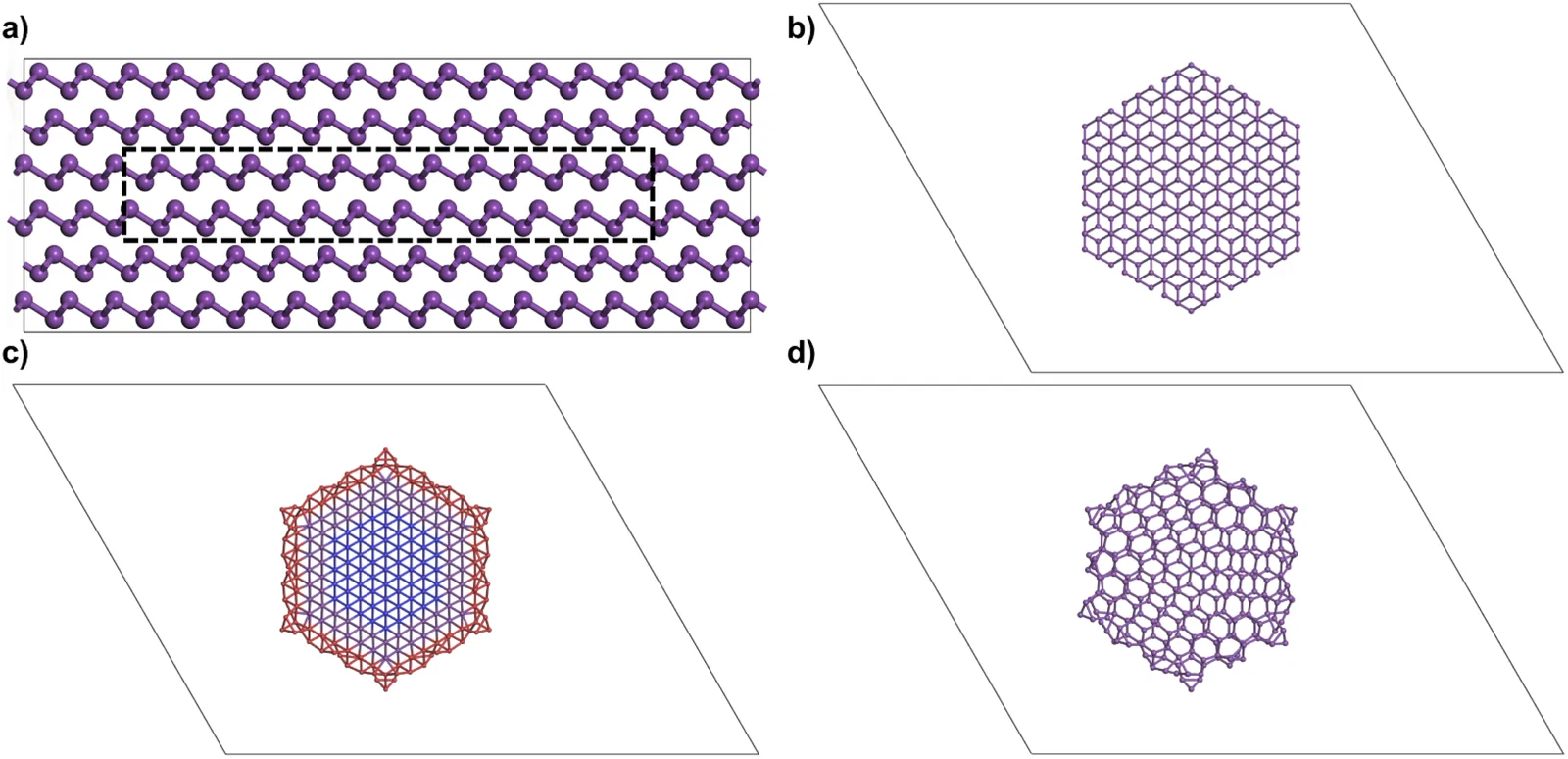
Atomistic models of bilayer bismuth flakes. (a) A ZY plane view of the 20 × 16 × 2 supercell of Bi I used as the starting structure. The broken lines indicate one flake. (b) An XY plane view of the 290-atom for the non-rotated and non-optimized bilayer bismuth flake. (c) An XY plane view of the optimized, non-rotated bismuth bilayer flake. The flake's core in blue, the flake's edge in red. (d) An XY plane view of the optimized structure of the flake with a rotation of 8.0° between layers.

Our strategy focused on the 0° to 20.0° interval as the primary region for investigation. Within this region, we sampled with high-resolution angles between 0.0° and 1.0° (using 0.25° increments) to clearly register the subtle early moiré pattern onset and potential low-angle effects. Above 1.0°, sampling density was reduced but selectively increased with supplemental points clustered about regions of interest, namely the 1–4° span (1.0°, 1.5°, 2.0°, 2.5°, 3.0°, 3.5°, 4.0°), the 9–11° span (9.0°, 9.5°, 10.0°, 10.5°, 11.0°), and the 19–21° span (19.0°, 19.5°, 20.0°, 20.5°, 21.0°). This targeted densification aimed to investigate potential local maxima in eDoS. Finally, extended coverage up to 30.0° was included to capture the asymptotic behavior as the twist-angle increases. This combined approach of focused densification around points of interest and extended coverage ensured a characterization of the twist-angle dependence.

The geometry optimization (GO) process was conducted using the Broyden–Fletcher–Goldfarb–Shanno (BFGS) algorithm.^21–24^ The convergence thresholds were 1 × 10^−5^ Ha for energy, 2 × 10^−3^ Ha Å^−1^ for maximum force, and 5 × 10^−3^ Å for maximum displacement. During relaxation, rotation axes were held fixed along the *z*-axis to conserve the desired twist-angles. The stable structure was verified through harmonic vibrational analysis, confirming the nonexistence of imaginary frequencies below 10 cm^−1^. The optimized structure of the TBB with a rotation of 8.0° between layers can be seen in Fig. 1d.

Each of the resulting structures is composed of three distinct regions, as illustrated in Fig. 2. At the center is a crystalline-like core with a diameter of approximately 17 Å (∼10 atomic layers), represented by blue atoms. The outermost region, surrounding the core, is a disordered edge that is ∼5 Å thick (∼3 atomic layers), shown as red atoms. Separating these two is a transition region of at least two atomic layers (purple atoms) that is neither fully crystalline nor disordered. This composite structure results in an overall flake geometry with a maximum and minimum diameter of 41 Å and 35 Å, respectively. To visualize the local topology within these regions, the bonding network was determined using a distance-based cutoff criterion, where all Bi–Bi pairs within a radius of 3.6 Å are considered bonded. Based on this connectivity, the local motifs are color-coded: cyan linkages indicate triangular motifs, green bonds highlight quadrilateral motifs (deformed squares), and yellow bonds indicate deformed pentagonal rings.

**Fig. 2:**
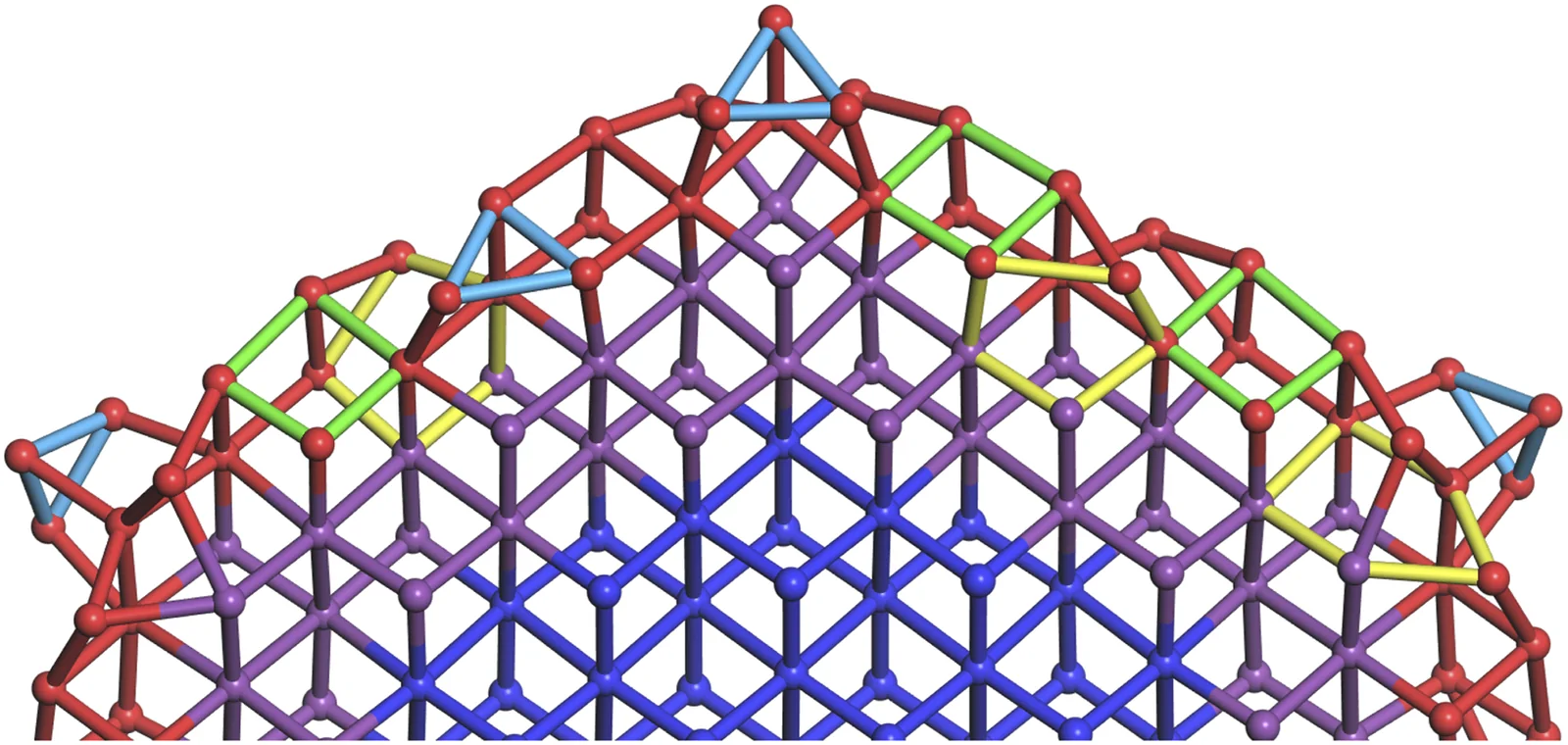
Optimized atomic structure of the flake. Core and edge atoms are represented by blue and red spheres, respectively. Colored bonds indicate specific geometric motifs: triangular (cyan), square (green), and pentagonal (yellow). The bonding network was determined using a distance-based cutoff criterion, where all Bi–Bi pairs within a radius of 3.6 Å are considered bonded.

Electronic structure calculations were performed using DMol^3^ contained in the Materials Studio Suite.^25^ Parameters adopted for the density functional theory consisted of the Vosko–Wilk–Nusair exchange–correlation functional (VWN-LDA),^26^ a double numerical plus polarization basis set, along with a density functional semi-core pseudopotential (DSPP) treatment for the cores. The calculations used a *Γ*-centered 4 × 4 × 1 *k*-point sampling, a 6.0 Å real-space orbital cutoff for the wave-function basis set, and a 1 × 10^−6^ Ha energy tolerance.

In addition, we used an integration grid with octupolar angular momentum fitting functions. Lastly, the eDoS was obtained by means of single-point energy calculations on optimized structures. Vibrational densities of states (vDoS, *F*(*ω*)) were obtained using the finite displacement method with a 0.002 Å displacement step to construct the dynamical matrix.

## 3 Results and discussion

### 3.1 Structural analysis

Characterization of the bismuthene structures was accomplished through the extensive study of the optimized flakes' geometry. Correlation,^27^ a computer code developed by our group, was used for this purpose. This tool enabled the calculation of two key functions for structural description: the pair distribution function (PDF, *g*(*r*)), and the plane-angle distribution function (*F*(*θ*)).

The pair distribution function, gives information about the probability of finding an atom at a distance *r* away from a selected reference atom, providing a quantitative description of the atomic density as a function of distance. The plane-angle distribution function, on the other hand, is crucial for understanding the orientation of bonds and the local arrangement within the structure, as it quantifies the distribution of angles formed by neighboring atoms. The results obtained using this analysis are discussed in what follows.

Following the GO process, the flake exhibits two distinct regional behaviors. The core of the flake undergoes a rigid-like compression relative to the bulk Bi I crystal structure (Fig. 1a), an effect that is manifested in the comparison of the PDF in Fig. 3a. A 3.4% compression in volume of the parent Bi I structure produces an exact replica of first and second neighbor interatomic distances in the flake's core atoms. The third and fourth nearest-neighbor interatomic distances are also compressed and matched with an error less than 4%, as depicted in Fig. 3b.

**Fig. 3:**
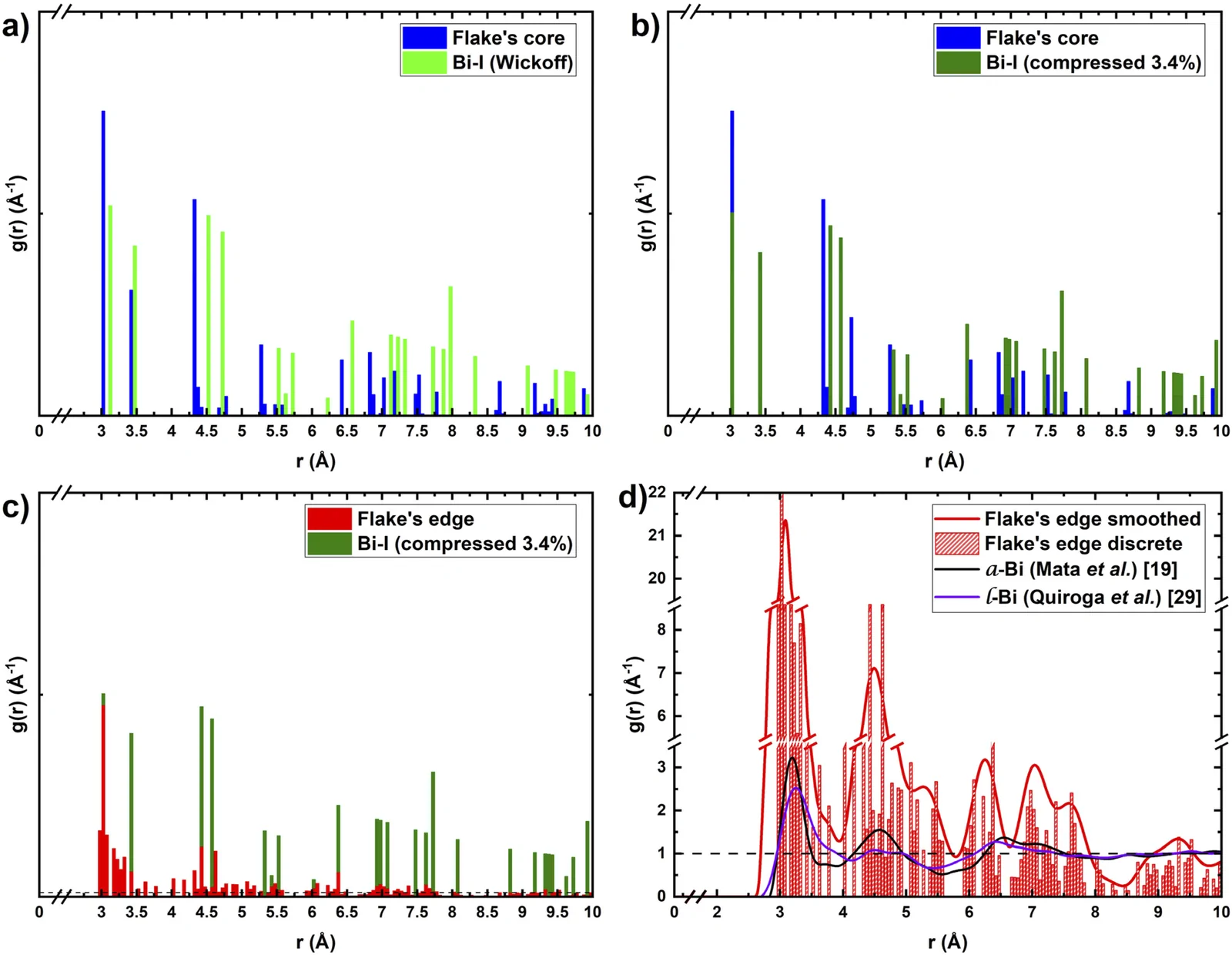
Pair distribution functions of the bilayer bismuth flakes. (a) PDF of the flake's core atoms compared to the bulk Bi I phase, highlighting the crystalline nature of the core. (b) Comparison of the flake's core PDF with that of a 3.4% compressed bulk Bi I structure, revealing the core's compressed state. (c) PDF of the flake's edge atoms compared to compressed Bi I, showing significant deviation from the bulk crystal structure. (d) Smoothed and discrete PDF of the flake's edge (in red), compared with amorphous (in black)^19,28^ and liquid bismuth (in purple),^29^ to illustrate the disordered nature of the flake's edge.

Optimization led to a result where the flake's edge became heavily disordered, almost amorphous-like (Fig. 1d), a state clearly characterized by its PDF (Fig. 3c), the edge PDF deviates significantly from the crystalline structure. Then we compared the flake's edge PDF with that of amorphous and liquid bismuth^19,28,29^ as shown in Fig. 3d, finding wide delocalized peaks characteristic of disordered materials. This finding provides compelling evidence for the disordered nature of the flake's edge. A further analysis reveals that the flake's edge PDF lacks the characteristic shoulder between 3.5 Å and 4.0 Å, which is a hallmark of liquid bismuth,^28,29^ definitively ruling out a molten state. Furthermore, the edge of the flake shows a 3.9% compression compared to both the amorphous and liquid bismuth, suggesting a unique and highly strained structural arrangement.

Pair distribution functions have been shown to be effective in the description of significant structural variations across the bismuth flake; the core's compression and the edge's disordering, provides information only about interatomic distances. To gain a more complete picture of the local atomic arrangement and validate these findings, it is essential to analyze the bond angles of the geometry relaxed structures. In Fig. 4, we show the results of *F*(*θ*) for the untwisted flake (0.0° twist-angle).

**Fig. 4:**
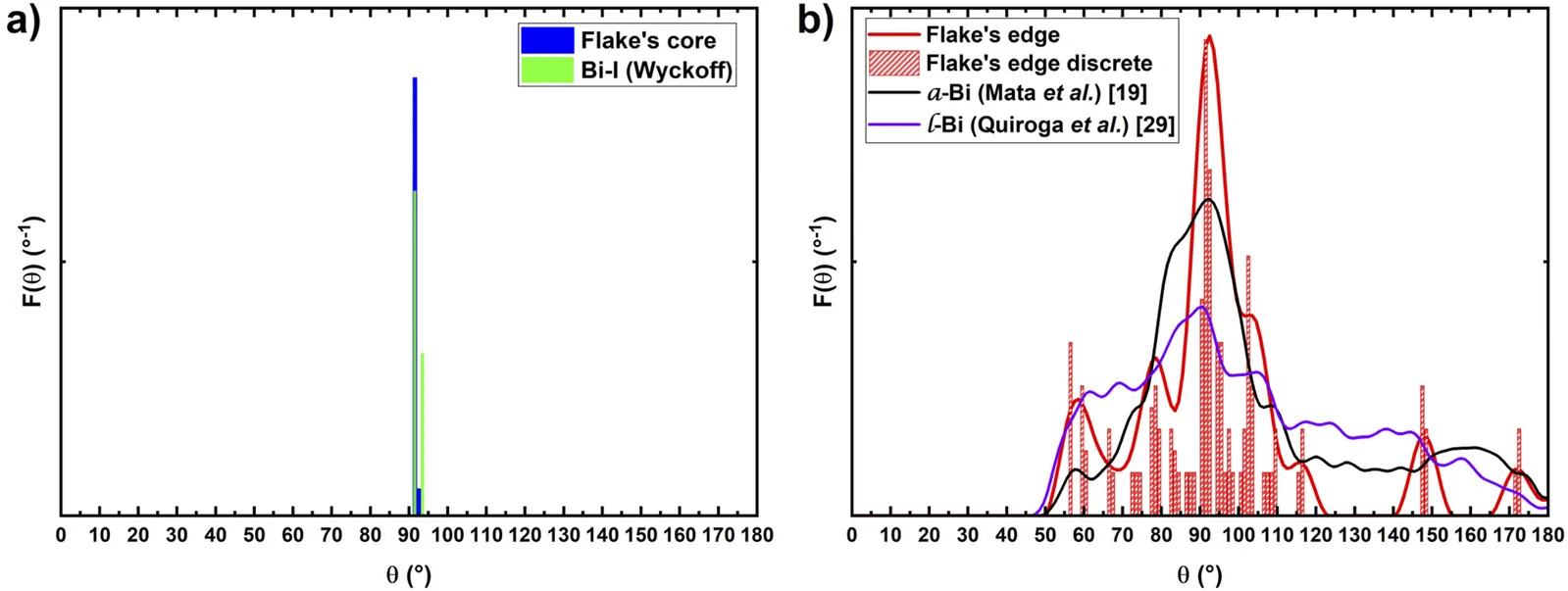
Plane-angle distribution (*F*(*θ*)) analysis for 0.0° twist-angle. (a) *F*(*θ*) of the flake's core (blue) contrasted with characteristic peaks of crystalline Bismuth I (green), demonstrating the preservation of the core's ordered structure. (b) Smoothed and discrete *F*(*θ*) of the flake's edge (red) compared to amorphous (black)^19,28^ and liquid bismuth (purple).^29^ The broad, featureless distribution of the edge confirms its disordered, non-crystalline state.

### 3.2 Vibrational analysis

Analysis of the *F*(*θ*), reveals distinct differences between the flake's core and edge. As shown in Fig. 4a, the flake's core *F*(*θ*) exhibits two peaks at 91.5° and 92.5°, closely mirroring the crystalline Bi I structure's main peak at 91.5° and secondary peak at 93.5°. This close correspondence confirms that the core preserves the ordered, symmetrical lattice of the parent Bi I crystal. In contrast, the flake's edge presents a broad, prominent peak at 90° and a secondary broad peak at 57° (Fig. 3b). This multimodal distribution indicates a breakup of the hexagonal lattice into a random network of deformed triangles (57°), squares (90°), and pentagons (116°). Such peak broadening and geometric diversity confirm the disordered nature of the edge, aligning with the PDFs results.

### 3.3 Electronic density of states

In order to investigate the relevance of the relativistic spin–orbit coupling, referred to frequently, we undertook the task of studying its effect on the electronic density of states of bismuth. Our study was motivated by the results reported by Cava and collaborators^30^ where they find that for the square structures of Bi–Li, the effect of the spin–orbit coupling manifests itself for energies below the Fermi level, and therefore does not affect any property related to the electron states in the vicinity of the Fermi energy, like superconductivity. The sample studied has the Wyckoff structure, and is stable at room temperature and at atmospheric pressure. We used a supercell smaller than the size of the flake for this enquiry. Our results, depicted in Fig. 5, indicate that, in fact, the influence of the spin–orbit interaction in the electronic structure of bismuth appears predominantly for energies below *E*_F_. The value for the density of states is practically the same at the Fermi level. So the estimations reported in this paper do not consider the effect of the spin–orbit coupling.

**Fig. 5:**
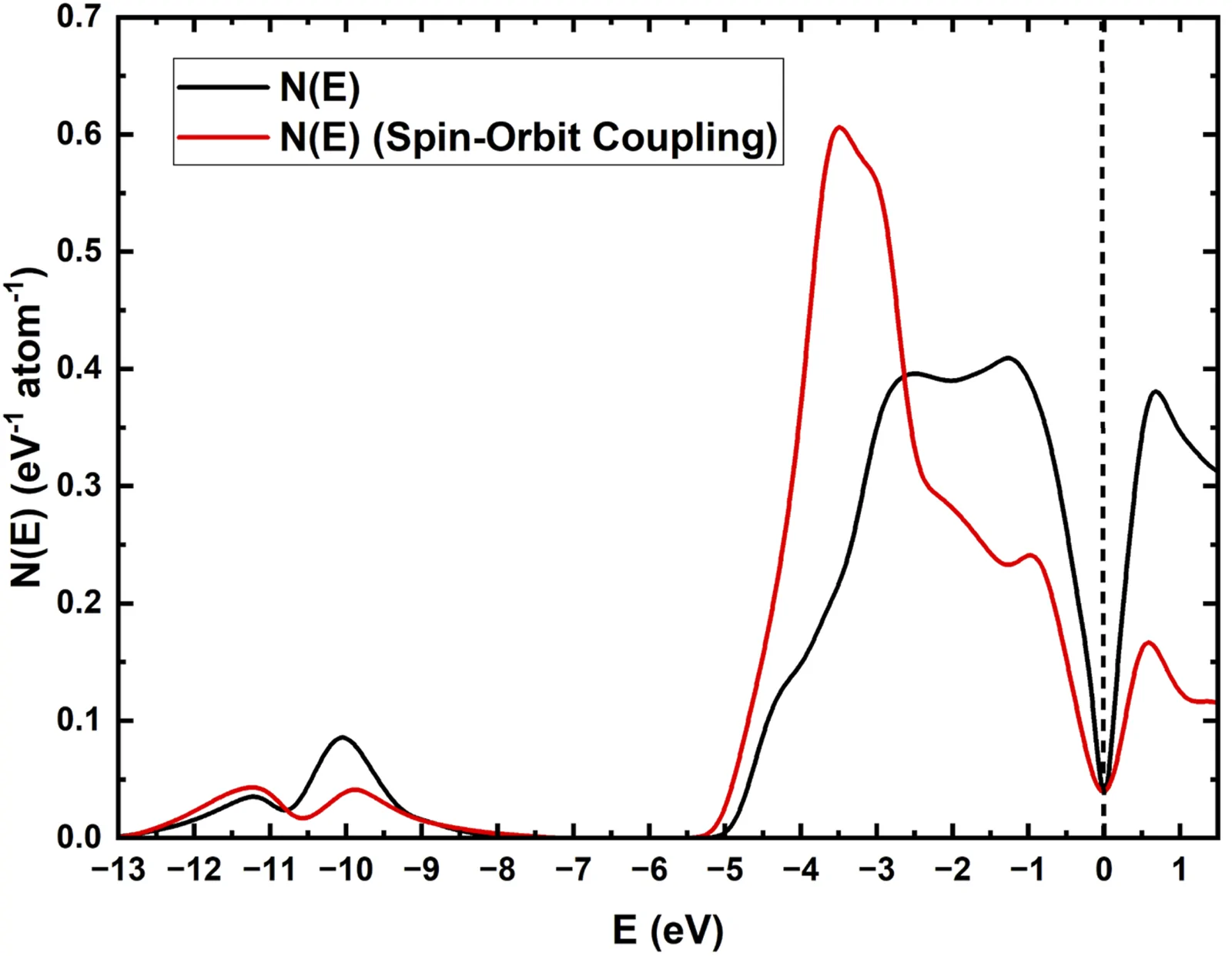
Comparison of the bismuth-Wyckoff electronic density of states, illustrating calculations performed both excluding (black line) and including (red line) relativistic spin–orbit coupling effects. The electronic densities of states around the Fermi energy are very similar and therefore spin–orbit coupling is not relevant for our estimated results.

The electronic density of states for the TBB samples reveals a complex structure comprised of two major energy bands (Fig. 6). The first band, spanning from approximately −15 eV to −8 eV, is primarily composed of deeper-lying Bi 6s and 6p states. Features within this region, such as the distinct peaks around −12 eV and −10 eV, are attributed to localized atomic orbitals. In Fig. 6, we can observe the variation across all studied twist angles, both in the flake's core and in the flake's edge.

**Fig. 6:**
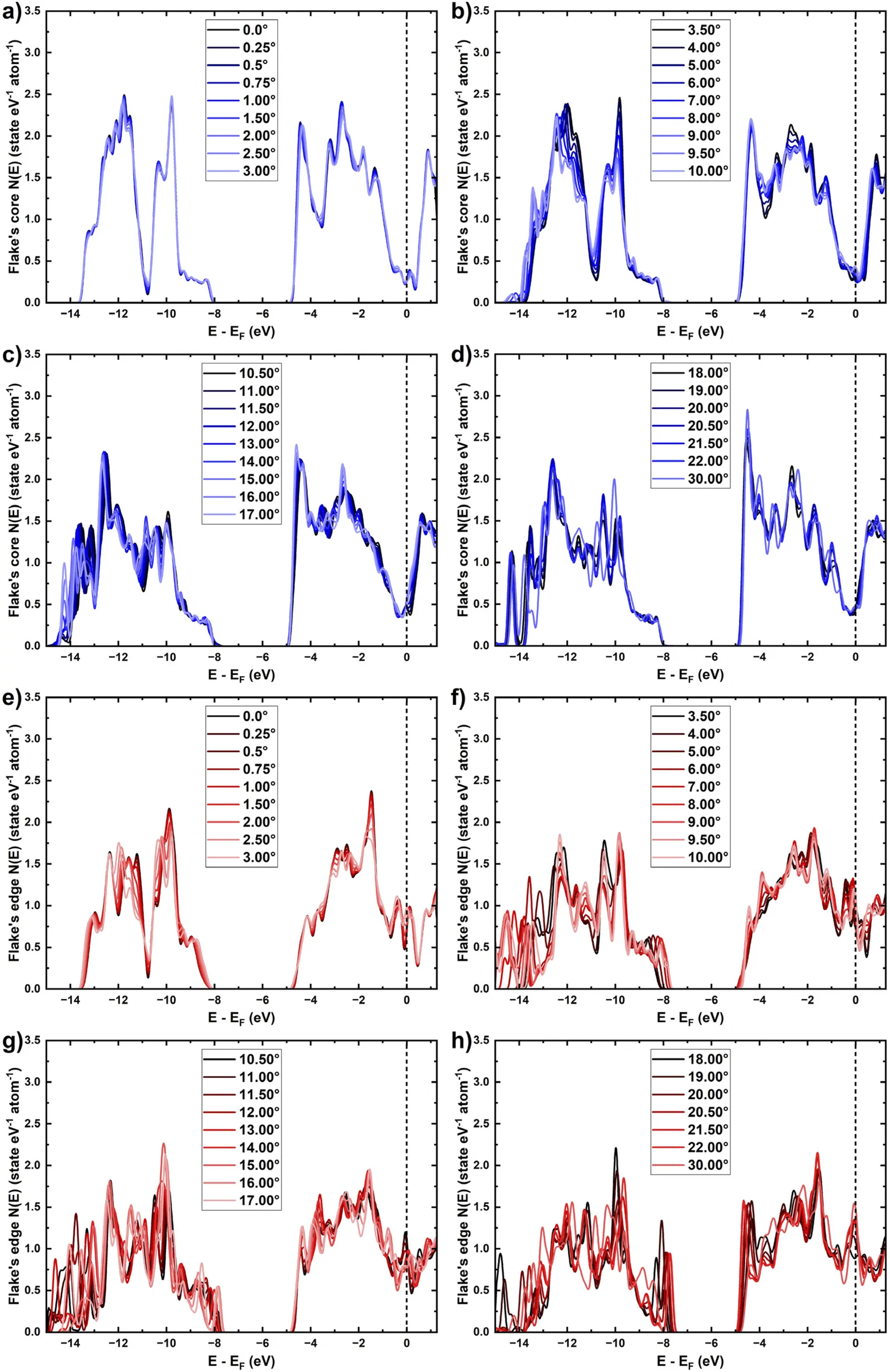
Electronic density of states for the TBB. Panels (a)–(d) show the eDoS obtained for the core region of the flake, grouped by angular range: (a) 0.0° ≤ *θ* ≤ 3.0°, (b) 3.5° ≤ *θ* ≤ 10.0°, (c) 10.5° ≤ *θ* ≤ 17.0°, and (d) 18.0° ≤ *θ* ≤ 30.0°. Panels (e)–(h) present the corresponding eDoS for the edge region of the flake, using the same angular grouping as (a)–(d).

The second band, narrower, expands from approximately −5 eV to the Fermi level (*E*_F_), and contains the electronic states crucial for this material's electronic properties. This region is highly sensitive to the interlayer twist-angle in the flake's edge region (Fig. 6e–h) and is dominated by Bi 6p orbitals in the whole flake (Fig. 7). The non-zero electronic density of states at the Fermi level, *N*(*E*_F_), indicates a metallic-like character. These two primary bands are separated by a wide energy gap between −8 eV and −5 eV.

**Fig. 7:**
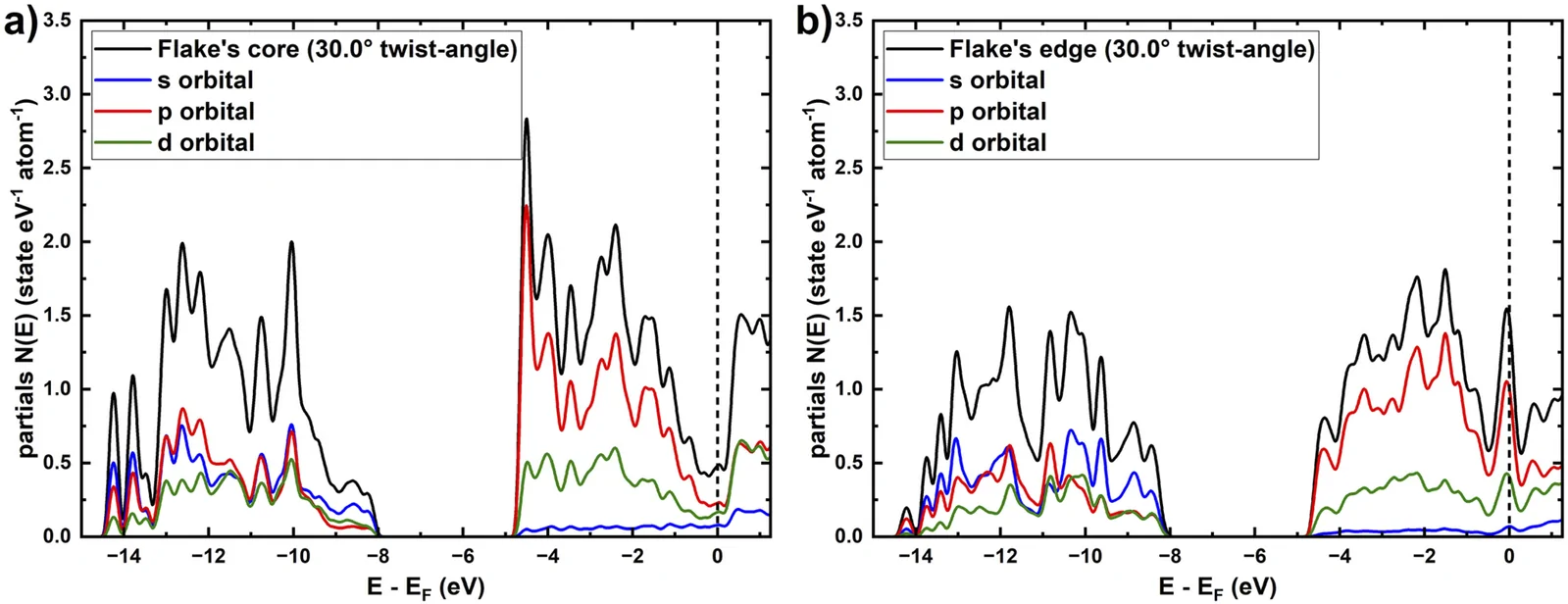
Partial and total electronic density of states for the twisted bismuth bilayer at a twist-angle of 30°. (a) *N*(*E*) for the flake's core. (b) *N*(*E*) for the flake's edge. The total *N*(*E*) is shown in black. Orbital-projected contributions from the Bi 6s, 6p, and 6d orbitals are displayed in blue, red, and green, respectively.

The significant difference in structural topology and confinement between the flake's core and the flake's edge requires analyzing these regions separately to understand the electronic properties of the system.

Analysis of the flake's core region (Fig. 6a–d), reveals a surprising twist-angle invariance. The electronic structure is dominated by a non-zero valley centered at the Fermi level, confirming the semi-metallic character of the core region. Crucially, the magnitude of *N*(*E*_F_), shows negligible variation across the entire range of the studied angles (Fig. 8). This invariance, is observed even around the previously reported small magic-angle of 0.5°,^13^ leading us to conclude that the states contributing to the Fermi surface of the flake's core are immune to slight variations in the moiré patterns for the entire range.

**Fig. 8:**
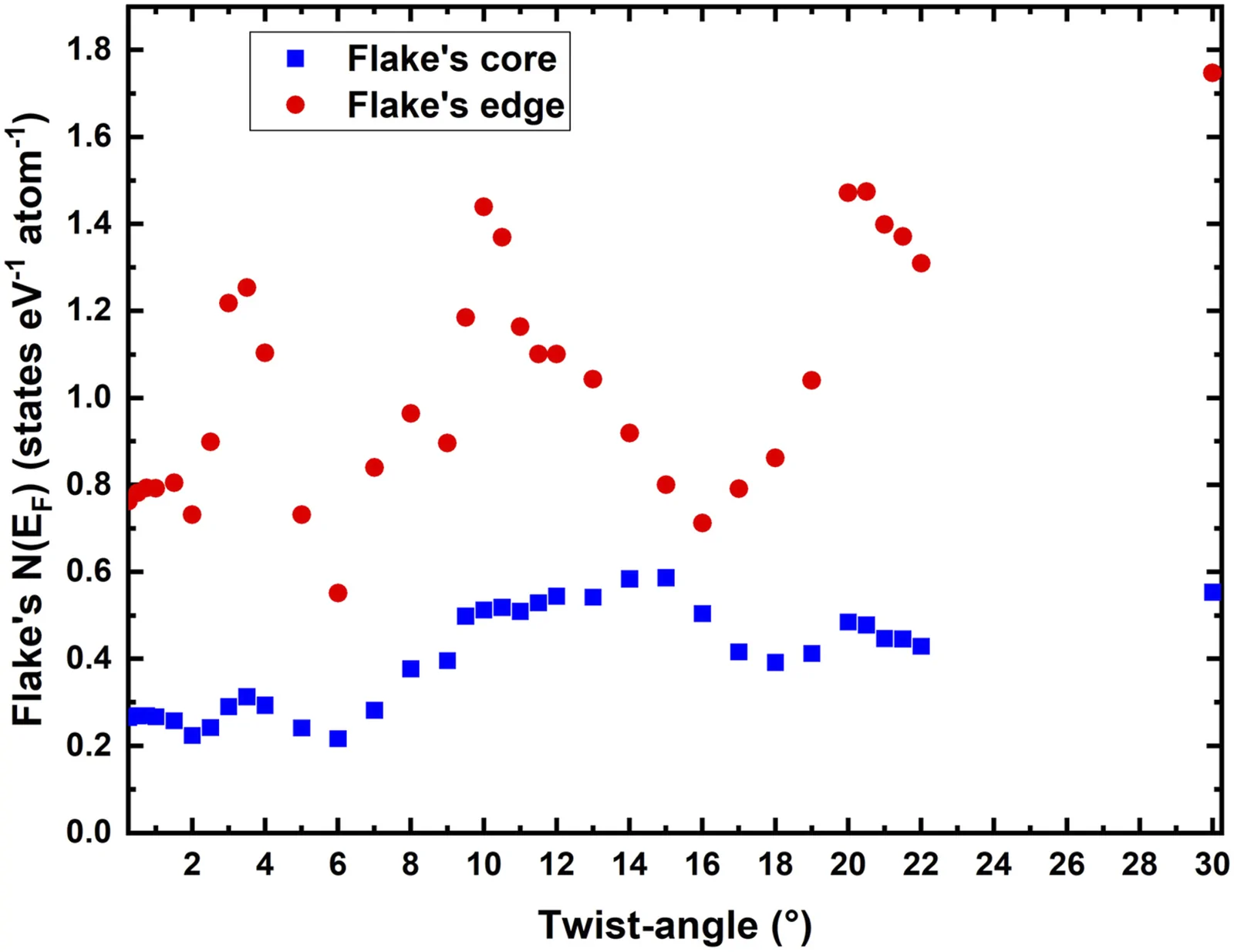
The electronic density of states at the Fermi level, reveals a striking contrast between the flake's core (blue squares) and its edge (red circles). The core exhibits a relatively invariant behavior with a single broad maximum of 0.59 states eV^−1^ atom^−1^ at *θ* = 15.0°. In contrast, the edge displays a highly oscillatory, non-monotonic *N*(*E*_F_) across the studied range, featuring four distinct maxima at 3.5°, 10.0°, 20.5°, and 30.0°.

The electronic density of states of the flake's edge (Fig. 6e–h) presents a different picture; it is characterized by the presence of a sharp peak at the Fermi level. The presence of this peak of the flake's edge, a feature typically associated with energy localized electronic states, indicates that the structural disorder, inherent to the flake boundary, reconfigures the electronic states in the vicinity of the Fermi level.

The persistence of a non-zero *N*(*E*_F_) in both the core and edge regions confirms that the system metallic-like character is maintained throughout the whole flake, although the energy states distribution near *E*_F_ is modulated by the geometric edge structure.

This structural and electronic dichotomy between the flake regions is quantitatively reflected in the *N*(*E*_F_) as a function of the interlayer twist-angle, presented in Fig. 8. The *N*(*E*_F_) of the flake's core (blue squares) displays a relatively invariant structure, peaking broadly at 0.59 states eV^−1^ atom^−1^ for *θ* = 15.0°. This electronic behavior for *N*(*E*) of the core is largely insensitive to the twist-angle, which strongly suggests that the flake's edge region is primarily responsible for the system-sensitive electronic phenomena, including the previously reported superconducting behavior effect at 0.5° for pristine TBB.^13^

In contrast, the *N*(*E*_F_) for the flake's edge (red circles) is highly sensitive to the rotation, showing a non-monotonic, oscillatory, dependence on the twist-angle, featuring four distinct local maxima at 3.5°, 10.0°, 20.5°, and 30.0°, representing a large 10-fold increase over the *N*(*E*_F_) of perfect crystalline Bi I structure, 0.15 states eV^−1^ atom^−1^.^13,19^ This behavior demonstrates that the edge's electronic properties are different from the TBB core, and are instead, dominated by the structural relaxations and the boundary states.

### 3.4 Superconducting and Debye temperatures

In what follows, a crude estimate of the effect of these changes on the possible superconducting transition temperature of the TBB will be presented. It should be clear that the method used does not pretend to be precise given the many approximations incorporated in the various versions of the theory of superconductivity, but should be considered approximate. Moreover, most of the theories have been developed with bulk materials in mind, and their application to lower dimensionality systems, like nanostructures, should be dealt with care. Nevertheless, the hope exists that the results reported herein will incite experimentalists and theoreticians to further investigate the possible influence that the topology of disordered structures may have on the *T*_c_, in the search for a room temperature superconductor.

Investigations of twisted van der Waals heterostructures, a field of research termed as twistronics, have effectively transformed the condensed matter physics dynamics ever since the magic-angle TGB phenomenon of superconductivity was discovered in 2018.^31^ The foundational principle is that a rotational misalignment between atomic layers generates moiré patterns which dramatically modifies the electronic structure. In particular, this situation can give rise to the existence of quasi-flat bands, where the electron group velocity is near zero.^32^ Flat bands energy-localize the electronic states, resulting in an extremely high electronic density of states around the Fermi level.

For the current work on the TBB, the high value of *N*(*E*_F_) established in the previous section, offers the onset condition for a possible superconducting phase. The following results detail the estimation of the Debye temperature *θ*_D_, as well as the *T*_c_, and explore the influence of the interlayer twist-angle as well as the structure defects on the flake's edge *versus* the core.

The initial step in the estimation of the possible *T*_c_ is to introduce the lattice dynamics. For the present work, the *F*(*ω*) was obtained using the Finite Displacement Method (FDM) (often referred to as the frozen-phonon method). The same parameters used for the *N*(*E*) electronic estimation were employed for the *F*(*ω*). To ensure computational stability and accuracy in obtaining the Hessian matrix, a finite difference displacement of 0.005 Å was used. Due to the supercell nature of the calculation in the FDM, the *F*(*ω*) represents the *q* = 0 (Gamma point) phonons. It would be desirable to include phonons beyond the Gamma point, but given the phenomenological nature of our approach this approximation is considered adequate. Subsequently, the Debye frequency (*ω*_D_) and the Debye temperature (*θ*_D_) were obtained from the resulting *F*(*ω*) using the formula proposed by Grimvall:^33^
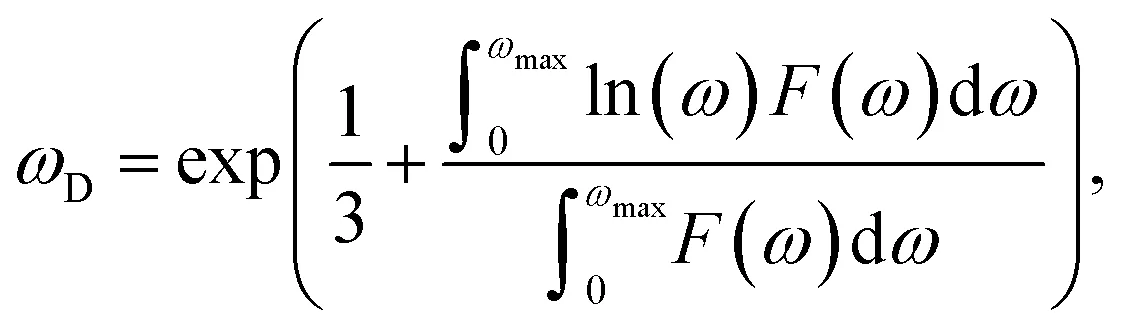
and
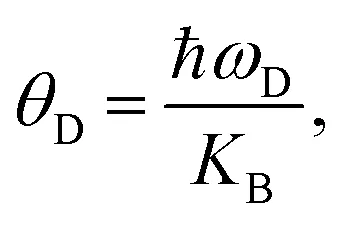
where *ħ* is the reduced Planck constant, and *K*_B_ is the Boltzmann constant.

The *θ*_D_ exhibits a non-monotonic dependence on the interlayer twist-angle, as illustrated in Fig. 8. Within the determined range of 0.0° to 10.0°, *θ*_D_ shows a periodic variation, and it was surmised that *θ*_D_ maintains this oscillation for larger angles. The largest value was 103 K for a twist-angle of 2.0°, and the lowest was 96.5 K for a twist-angle of 9.0°. This angular dependence suggests that the moiré pattern strongly modulates the lattice's vibrational properties.

To predict *θ*_D_ across the full angular spectrum and to quantify this periodic relationship, the data was successfully modeled using a sine fitting function. The resulting fit provided excellent statistical agreement, yielding an *R*^2^ value of 0.98 and a *χ*^2^ value of 0.08. The functional form of the fit is:
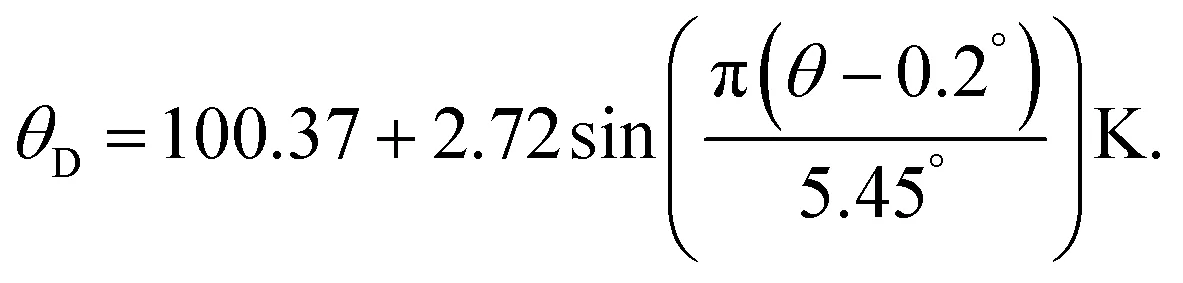


This oscillation in *θ*_D_, when coupled with the calculated *N*(*E*_F_) (Section 3.2), leads to an angle-dependent relation of the possible superconducting transition temperature.

To estimate the superconducting transition temperature, we assume that the Debye temperature has the same value at any given twist-angle for the crystalline-like flake's core and the disordered-like flake's edge. This approach is justified by comparing several allotropes of bismuth. The *θ*_D_ of bulk crystalline bismuth Bi I is 112 K, Bi II is 115.5 K, Bi III is 96.4 K, and Bi IV is 102.1 K,^34–37^ while for amorphous bismuth it has a value of 100 K.^19,38^ This small difference between several structures suggests that *θ*_D_ is not highly sensitive to the changes in atomic structure. Since the primary distinction between our flake's core and flake's edge is the loss of translational symmetry rather than local bond strength (compression of 4% for the core and 3.9% for the edge), we posit that their intrinsic *θ*_D_ values should be similarly close. Therefore, for subsequent estimations of the *T*_c_, the same angle-dependent *θ*_D_ derived above, is applied to both the crystalline-like core and the disordered-like edge regions.

In order to quantitatively estimate the *T*_c_ of the TBB system, we employ the phenomenological Mata–Valladares approach.^19^ It is a well-proven method for the calculation of *T*_c_ estimated from the computational evaluation of the electronic and vibrational properties. We selected this approach based on its past successes in estimating and predicting *T*_c_ for various allotropes and phases of bismuth,^13,19,36,39–41^ and based on the fact that the present theories of superconductivity rely on educated approximations that also makes them somewhat imprecise. Moreover, the application of some of the current approaches to calculating the superconducting transition temperatures to nanoflakes may be questionable. This approach takes the *N*(*E*_F_) values obtained in Section 3.2 and the angle-dependent *θ*_D_ values obtained in this section, and 0.53 mK, the experimental value of *T*_c_ for Bi I,^42^ to compute the superconducting transition. The resulting *T*_c_s are shown in Fig. 10. The *T*_c_ obtained shows a very large contrast between the edge and core regions of the flake, as anticipated, in view of the resulting electronic structure behavior for the *N*(*E*) (Fig. 8).

The *T*_c_ for the flake's core manifests a relatively bounded dependence on the twist-angle, reaching a maximum value of 4.7 K at a twist-angle of 15.0°. This observed behavior fits well with the established model of phonon-mediated (BCS-type) superconductivity in crystalline allotropes of bismuth.^43–45^

In contrast, the *T*_c_ of the edge of the flake shows a strong oscillatory and non-monotonic character. It exhibits four clear local maxima at precisely the same locations as the peaks in *N*(*E*_F_) obtained in the edge region analysis (Fig. 8). The *T*_c_ culminates in an interesting and provoking maximum of 38 K at a twist-angle of 30.0°. As expected, the correlation between the *T*_c_ maxima (Fig. 10) and the *N*(*E*_F_) peaks (Fig. 8) strongly suggests a major dependence on the *N*(*E*_F_), with a minor influence of the variation in *θ*_D_ (Fig. 9). This strong dependence on the twist-angle rotation reveals that the high *T*_c_ in the flake's edge is a result of the energy localization of the electronic states at the borders of the flake.^46^

**Fig. 9:**
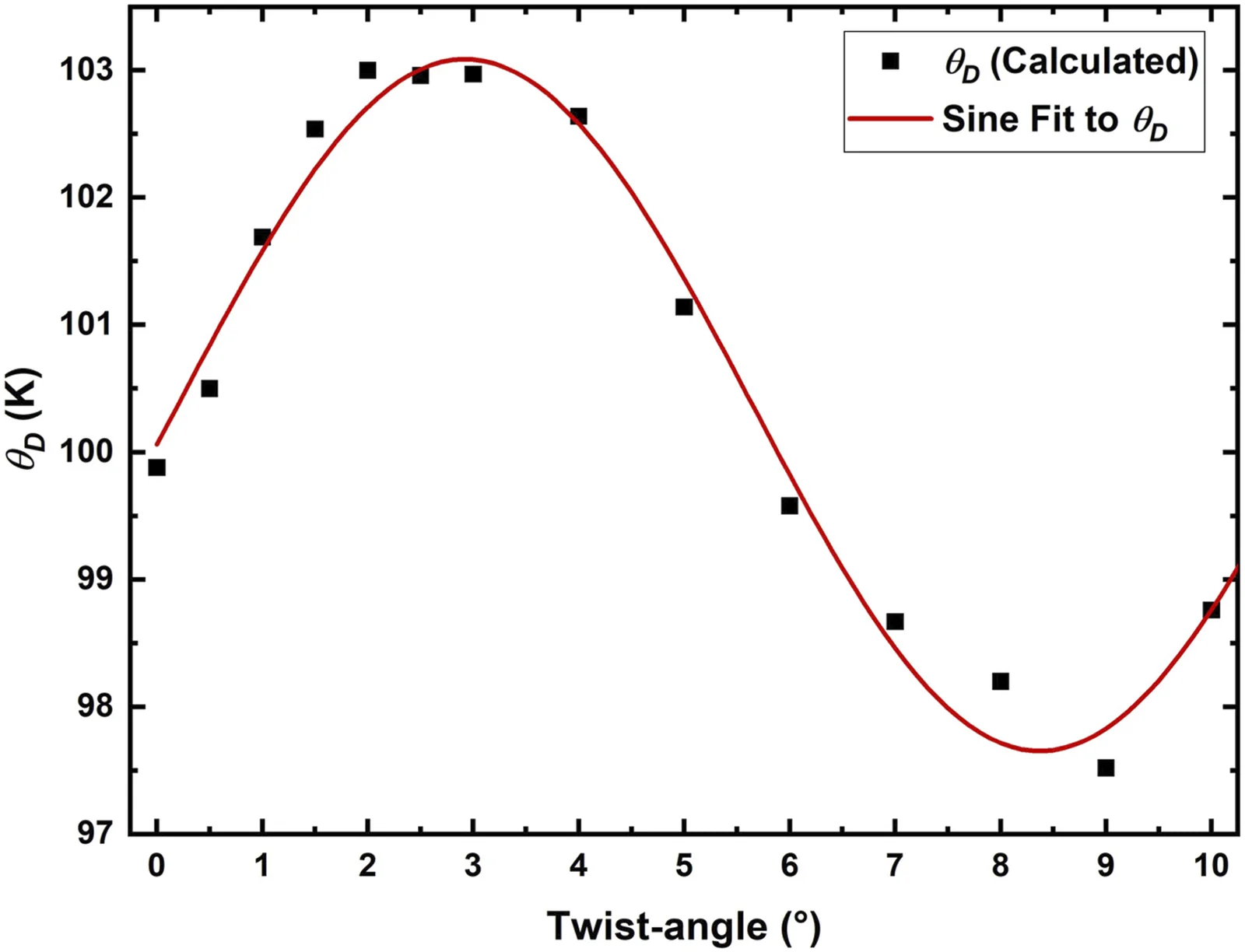
Debye temperatures (*θ*_D_) (in Kelvin) for the twisted bismuth bilayer system, calculated from the vibrational density of states *via* the Grimvall method, shown for twist angles ranging from 0.0° to 10.0°. The values are represented by black squares. The data shows a periodic oscillation, which is well-described by the sine fit (red line), highlighting the influence of the moiré patterns on the Debye temperature. This sinusoidal behavior was assumed to occur for angles larger than 10°.

**Fig. 10:**
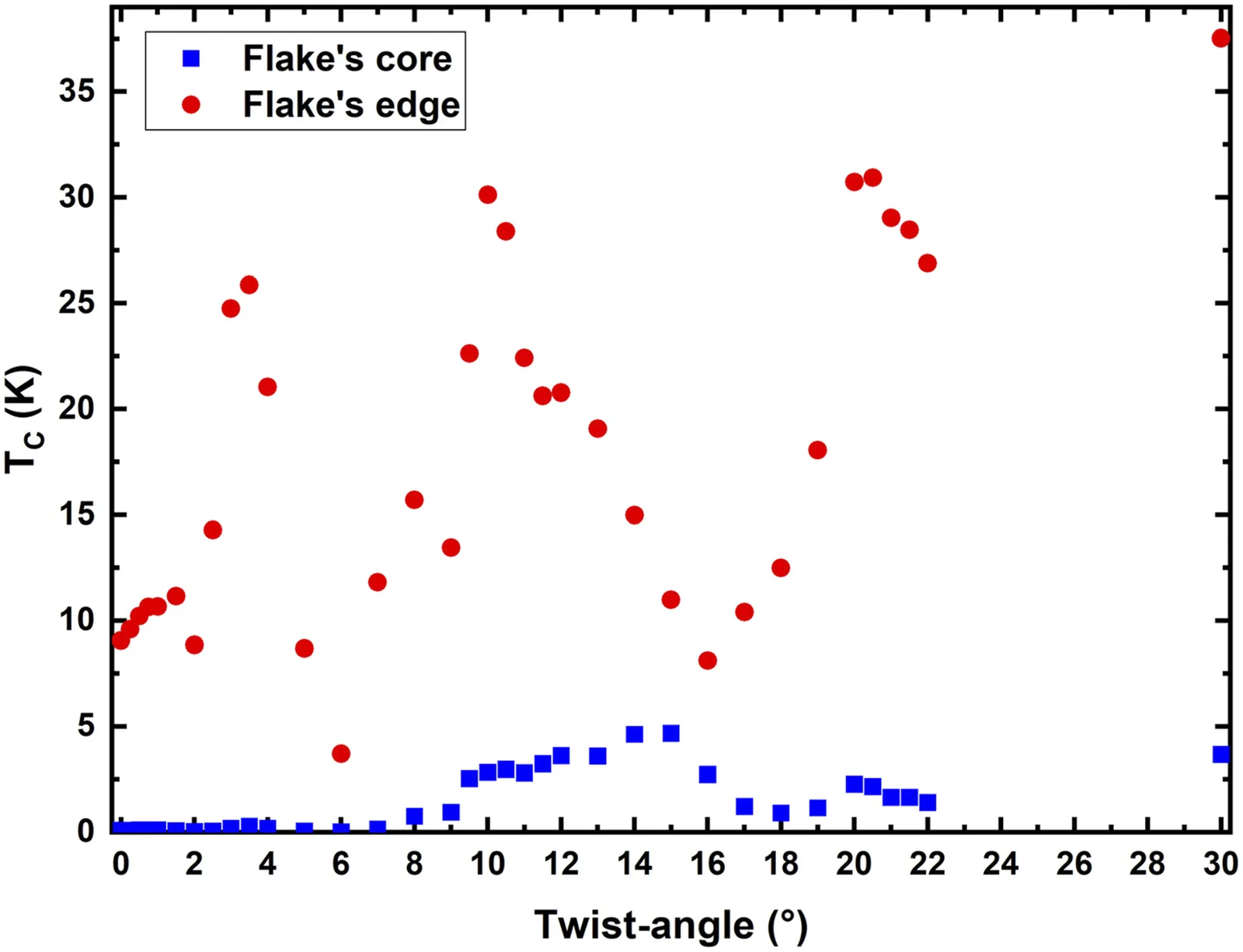
Estimated superconducting transition temperature, *T*_c_, for the twisted bismuth bilayer system, comparing the flake's core (blue) and the flake's edge (red) regions across the full angular range. The *T*_c_ values for the core follows a smooth trend for the entire range of studied twist-angles. In contrast, the edge region exhibits a highly oscillatory *T*_c_, culminating in a remarkable maximum value of 38 K at a twist angle of 30.0°.

Our work identifies two principal mechanisms for enhancing the superconducting transition temperature in twisted bilayer systems: twist-angle engineering and disorder, the latter being a well-documented route to higher *T*_c_.^47–49^ This finding is consistent with a growing consensus that for 2D materials disorder can enhance superconductivity.^50–54^

## 4 Conclusions

This density functional theory work reveals the role of structural heterogeneity in modulating the electronic properties of the twisted bismuth bilayers. Our findings confirm a clear relation between the flake's disorder, the electronic structure, and a potential high-temperature superconductor.

The full geometry optimization confirms a structural dichotomy within the flake: core *vs.* edge. The core maintains a compressed, crystalline-like structure, while the flake's edge undergoes significant relaxation, adopting a disordered-like configuration with the appearance of deformed triangular, quadrangular, and pentagonal atomic structures caused by the relaxation at the borders of the flake. This structural contrast is central to the electronic properties of the material.

Because of the structure dichotomy, the electronic density of states at the Fermi level shows a dual behavior. The crystalline-like core maintains a semi-metallic character with *N*(*E*_F_) showing a minimal variation for the whole range of twist-angles. Its profile is bounded with a peak value of 0.59 states eV^−1^ atom^−1^ at *θ* = 15.0°. The disordered-like edge, in contrast, is characterized by an oscillatory *N*(*E*_F_) profile, driven by localized peaks in the electronic density of states.

The estimated superconducting critical temperatures reveal a contrast in behavior. The core exhibits a conventionally low *T*_c_, peaking at 4.7 K for *θ* = 15.0°. The edge *T*_c_ exhibits a non-monotonic trend, reaching a maximum of 38 K for *θ* = 30.0°. The perfect one-to-one correlation between the *T*_c_ and the *N*(*E*_F_) peaks confirms that the structural disorder at the edge, when used within the BCS framework, is the driver of the *T*_c_ enhancement.

In summary, this work establishes edge-angle engineering as a new design principle for creating superconducting topologically enhanced twisted bilayered devices. The potential for a possible *T*_c_ enhancement at the flake's edge by 7 × 10^3^ times that of 0.53 mK for the crystalline Bi I, suggests that structural defects and edge effects, can be leveraged to drive possible emergent properties. Our results seem to indicate that the generation of new structures either by high pressure, or by twisting bilayer, or by generating new disordered structures, at the borders of flakes, is one way to go to search for new high *T*_c_ superconductors.

## Author contributions

This research was conceived and designed by I. R. and A. A. V., with input from R. M. V., A. V., D. H.-R. and F. B. Q. All simulations were performed by I. R. All authors contributed to the discussion and analysis of the results. I. R. and A. A. V. wrote the initial draft of the manuscript, which was subsequently enriched and approved by all co-authors for publication.

## Conflicts of interest

The authors declare no conflict of interest in this work.

## Data Availability

The datasets used and analyzed during the current study are available from the corresponding author on reasonable request.

## References

[cit1] Ersan F., Aktürk E., Ciraci S. (2016). Stable single-layer structure of group-V elements. Phys. Rev. B.

[cit2] Tyner A. C., Goswami P. (2023). Spin-charge separation and quantum spin Hall effect of β-bismuthene. Sci. Rep..

[cit3] Phu T. K. C., Hong W. T., Han H., Song Y. I., Kim J. H., Roh S. H., Kim M.-C., Koh J. H., Oh B.-K., Kim J. Y., Chung C.-H., Lee D. H., Kim J. K. (2024). Conformal surface intensive doping of low-valence Bi on Cu_2_O for highly efficient electrochemical nitrate reduction to ammonia production. Mater. Today.

[cit4] Lopes dos Santos J. M. B., Peres N. M. R., Castro Neto A. H. (2007). Graphene Bilayer with a Twist: Electronic Structure. Phys. Rev. Lett..

[cit5] Yankowitz M., Chen S., Polshyn H., Zhang Y., Watanabe K., Taniguchi T., Graf D., Young A. F., Dean C. R. (2019). Tuning superconductivity in twisted bilayer graphene. Science.

[cit6] Pons R., Mielke A., Stauber T. (2020). Flat-band ferromagnetism in twisted bilayer graphene. Phys. Rev. B.

[cit7] Cantele G., Alfè D., Conte F., Cataudella V., Ninno D., Lucignano P. (2020). Structural relaxation and low-energy properties of twisted bilayer graphene. Phys. Rev. Res..

[cit8] Chowdhury E. H., Rahman Md. H., Bose P., Jayan R., Islam M. M. (2020). Atomic-scale analysis of the physical strength and phonon transport mechanisms of monolayer β-bismuthene. Phys. Chem. Chem. Phys..

[cit9] Gou J., Kong L., He X., Huang Y. L., Sun J., Meng S., Wu K., Chen L., Wee A. T. S. (2020). The effect of moiré superstructures on topological edge states in twisted bismuthene homojunctions. Sci. Adv..

[cit10] Cancès É., Garrigue L., Gontier D. (2023). Simple derivation of moiré-scale continuous models for twisted bilayer graphene. Phys. Rev. B.

[cit11] Uri A., De La Barrera S. C., Randeria M. T., Rodan-Legrain D., Devakul T., Crowley P. J. D., Paul N., Watanabe K., Taniguchi T., Lifshitz R., Fu L., Ashoori R. C., Jarillo-Herrero P. (2023). Superconductivity and strong interactions in a tunable moiré quasicrystal. Nature.

[cit12] Huang W., Zhu J., Wang M., Hu L., Tang Y., Shu Y., Xie Z., Zhang H. (2021). Emerging Mono-Elemental Bismuth Nanostructures: Controlled Synthesis and Their Versatile Applications. Adv. Funct. Mater..

[cit13] Rodríguez I., Valladares R. M., Hinojosa-Romero D., Valladares A., Valladares A. A. (2024). Superconductivity in Twisted Bismuth Bilayers. Adv. Phys. Res..

[cit14] Kowalczyk P. J., Belic D., Mahapatra O., Brown S. A., Kadantsev E. S., Woo T. K., Ingham B., Kozlowski W. (2012). Anisotropic oxidation of bismuth nanostructures: Evidence for a thin film allotrope of bismuth. Appl. Phys. Lett..

[cit15] Kokubo I., Yoshiike Y., Nakatsuji K., Hirayama H. (2015). Ultrathin Bi(110) films on Si ( 111 ) 3 × 3 − B substrates. Phys. Rev. B.

[cit16] Hirayama H. (2021). Nucleation and growth of ultrathin Bi films. Adv. Phys.:X.

[cit17] Dolle C., Oestreicher V., Ruiz A. M., Kohring M., Garnes-Portolés F., Wu M., Sánchez-Santolino G., Seijas-Da Silva A., Alcaraz M., Eggeler Y. M., Spiecker E., Canet-Ferrer J., Leyva-Pérez A., Weber H. B., Varela M., Baldoví J. J., Abellán G. (2023). Hexagonal Hybrid Bismuthene by Molecular Interface Engineering. J. Am. Chem. Soc..

[cit18] Liu X., Zhang S., Guo S., Cai B., A Yang S., Shan F., Pumera M., Zeng H. (2020). Advances of 2D bismuth in energy sciences. Chem. Soc. Rev..

[cit19] Mata-Pinzón Z., Valladares A. A., Valladares R. M., Valladares A. (2016). Superconductivity in Bismuth. A New Look at an Old Problem. PLoS One.

[cit20] Cucka P., Barrett C. S. (1962). The crystal structure of Bi and of solid solutions of Pb, Sn, Sb and Te in Bi. Acta Crystallogr..

[cit21] Broyden C. G. (1967). Quasi-Newton methods and their application to function minimisation. Math. Comput..

[cit22] Fletcher R. (1970). A new approach to variable metric algorithms. Comput. J..

[cit23] Goldfarb D. (1970). A family of variable-metric methods derived by variational means. Math. Comput..

[cit24] Shanno D. F. (1970). Conditioning of quasi-Newton methods for function minimization. Math. Comput..

[cit25] BIOVIA , Dassault Systèmes, DMol3, Materials Studio 2025, San Diego, 2025

[cit26] Vosko S. H., Wilk L., Nusair M. (1980). Accurate spin-dependent electron liquid correlation energies for local spin density calculations: a critical analysis. Can. J. Phys..

[cit27] Rodríguez I., Valladares R., Valladares A., Hinojosa-Romero D., Santiago U., Valladares A. A. (2021). Correlation: An Analysis Tool for Liquids and for Amorphous Solids. J. Open Source Softw..

[cit28] Quiroga F. B., Hinojosa-Romero D., Valladares A., Valladares R. M., Rodríguez I., Valladares A. A. (2022). The effect of negative pressures on the superconductivity of amorphous and crystalline bismuth. Sci. Rep..

[cit29] Quiroga F. B. , Rodríguez I., Hinojosa-Romero D., Valladares A., Valladares R. M. and Valladares A. A., The structure of a melt: The case of liquid bismuth, arXiv, 2025, preprint arXiv:2605.24234, 10.48550/arXiv.2605.24234

[cit30] Górnicka K., Gutowska S., Winiarski M. J., Wiendlocha B., Xie W., Cava R. J., Klimczuk T. (2020). Superconductivity on a Bi square net in LiBi. Chem. Mater..

[cit31] Cao Y., Fatemi V., Fang S., Watanabe K., Taniguchi T., Kaxiras E., Jarillo-Herrero P. (2018). Unconventional superconductivity in magic-angle graphene superlattices. Nature.

[cit32] Tian H., Gao X., Zhang Y., Che S., Xu T., Cheung P., Watanabe K., Taniguchi T., Randeria M., Zhang F., Lau C. N., Bockrath M. W. (2023). Evidence for Dirac flat band superconductivity enabled by quantum geometry. Nature.

[cit33] Grimvall G. , Thermophysical Properties of Materials, Elsevier, Amsterdam, 1999, pp. 89–94

[cit34] Gopinathan K. K., Padmini A. R. K. L. (1975). Ultrasonic studies on bismuth single crystals. Solid State Commun..

[cit35] Fischer P., Sosnowska I., Szymanski M. (1978). Debye-Waller factor and thermal expansion of arsenic, antimony and bismuth. J. Phys. C: Solid State Phys..

[cit36] Rodríguez I., Hinojosa-Romero D., Valladares A., Valladares R. M., Valladares A. A. (2019). A facile approach to calculating superconducting transition temperatures in the bismuth solid phases. Sci. Rep..

[cit37] Shankar Narayana M., Gopi Krishna N. (2005). X-ray study of Bi, Sb and Bi_1–*x*_–Sb_*x*_ alloys. Phys. Status Solidi A.

[cit38] Komnik Yu. F. (1982). Electrical properties of fine crystalline and amorphous metal films, obtained by low temperature condensation (Review), Sov. J. Low Temp. Phys..

[cit39] Hinojosa-Romero D., Rodríguez I., Mata-Pinzón Z., Valladares A., Valladares R., Valladares A. A. (2017). Compressed Crystalline Bismuth and Superconductivity –An ab initio computational Simulation. MRS Adv..

[cit40] Valladares A. A., Rodríguez I., Hinojosa-Romero D., Valladares A., Valladares R. M. (2018). Possible superconductivity in the Bismuth IV solid phase under pressure. Sci. Rep..

[cit41] Hinojosa-Romero D., Rodriguez I., Valladares A., Valladares R. M., Valladares A. A. (2018). Possible superconductivity in Bismuth (111) bilayers. Their electronic and vibrational properties from first principles. MRS Adv..

[cit42] Prakash O., Kumar A., Thamizhavel A., Ramakrishnan S. (2017). Evidence for bulk superconductivity in pure bismuth single crystals at ambient pressure. Science.

[cit43] Bardeen J., Cooper L. N., Schrieffer J. R. (1957). Microscopic Theory of Superconductivity. Phys. Rev..

[cit44] Bardeen J., Cooper L. N., Schrieffer J. R. (1957). Theory of Superconductivity. Phys. Rev..

[cit45] Stewart G. R. (2017). Unconventional superconductivity. Adv. Phys..

[cit46] Arturo Sánchez-Sánchez J., Navarro-Espino M., Betancur-Ocampo Y., Barrios-Vargas J. E., Stegmann T. (2022). Steering the current flow in twisted bilayer graphene. J. Phys. Mater..

[cit47] Anderson P. W. (1959). Theory of dirty superconductors. J. Phys. Chem. Solids.

[cit48] Shier J. S., Ginsberg D. M. (1966). Superconducting Transitions of Amorphous Bismuth Alloys. Phys. Rev..

[cit49] Tsuei C. C. , Amorphous Superconductors, in Superconductor Materials Science: Metallurgy, Fabrication, and Applications, ed. S. Foner and B. B. Schwartz, Springer US, Boston, MA, 1981, pp. 735–756, 10.1007/978-1-4757-0037-4_12

[cit50] Peng J., Yu Z., Wu J., Zhou Y., Guo Y., Li Z., Zhao J., Wu C., Xie Y. (2018). Disorder Enhanced Superconductivity toward TaS_2_ Monolayer. ACS Nano.

[cit51] Vasiakin M. M., Mel’nikov A. S. (2025). Disorder-enhanced superconductivity in altermagnet-superconductor hybrids. Phys. Rev. B.

[cit52] Dubi Y., Meir Y., Avishai Y. (2008). Island formation in disordered superconducting thin films at finite magnetic fields. Phys. Rev. B.

[cit53] Sacépé B., Dubouchet T., Chapelier C., Sanquer M., Ovadia M., Shahar D., Feigel’man M., Ioffe L. (2011). Localization of preformed Cooper pairs in disordered superconductors. Nat. Phys..

[cit54] Lemarié G., Kamlapure A., Bucheli D., Benfatto L., Lorenzana J., Seibold G., Ganguli S. C., Raychaudhuri P., Castellani C. (2013). Universal scaling of the order-parameter distribution in strongly disordered superconductors. Phys. Rev. B.

